# Construction of disulfidptosis-based immune response prediction model with artificial intelligence and validation of the pivotal grouping oncogene c-MET in regulating T cell exhaustion

**DOI:** 10.3389/fimmu.2024.1258475

**Published:** 2024-01-26

**Authors:** Pengping Li, Shaowen Wang, Hong Wan, Yuqing Huang, Kexin Yin, Ke Sun, Haigang Jin, Zhenyu Wang

**Affiliations:** ^1^Department of Thyroid and Breast Surgery, The First People’s Hospital of Xiaoshan District, Xiaoshan Affiliated Hospital of Wenzhou Medical University, Hangzhou, Zhejiang, China; ^2^Neuromedicine Center, The University of Hong Kong-Shenzhen Hospital, Shenzhen, Guangdong, China; ^3^Department of General Surgery, Breast Surgery, The First Affiliated Hospital of Anhui Medical University, Hefei, China

**Keywords:** disulfidptosis, tumor immunity, prognosis prediction, artificial intelligence (AI), glioma

## Abstract

**Background:**

Given the lack of research on disulfidptosis, our study aimed to dissect its role in pan-cancer and explore the crosstalk between disulfidptosis and cancer immunity.

**Methods:**

Based on TCGA, ICGC, CGGA, GSE30219, GSE31210, GSE37745, GSE50081, GSE22138, GSE41613, univariate Cox regression, LASSO regression, and multivariate Cox regression were used to construct the rough gene signature based on disulfidptosis for each type of cancer. SsGSEA and Cibersort, followed by correlation analysis, were harnessed to explore the linkage between disulfidptosis and cancer immunity. Weighted correlation network analysis (WGCNA) and Machine learning were utilized to make a refined prognosis model for pan-cancer. In particular, a customized, enhanced prognosis model was made for glioma. The siRNA transfection, FACS, ELISA, etc., were employed to validate the function of c-MET.

**Results:**

The expression comparison of the disulfidptosis-related genes (DRGs) between tumor and nontumor tissues implied a significant difference in most cancers. The correlation between disulfidptosis and immune cell infiltration, including T cell exhaustion (Tex), was evident, especially in glioma. The 7-gene signature was constructed as the rough model for the glioma prognosis. A pan-cancer suitable DSP clustering was made and validated to predict the prognosis. Furthermore, two DSP groups were defined by machine learning to predict the survival and immune therapy response in glioma, which was validated in CGGA. PD-L1 and other immune pathways were highly enriched in the core blue gene module from WGCNA. Among them, c-MET was validated as a tumor driver gene and JAK3-STAT3-PD-L1/PD1 regulator in glioma and T cells. Specifically, the down-regulation of c-MET decreased the proportion of PD1+ CD8+ T cells.

**Conclusion:**

To summarize, we dissected the roles of DRGs in the prognosis and their relationship with immunity in pan-cancer. A general prognosis model based on machine learning was constructed for pan-cancer and validated by external datasets with a consistent result. In particular, a survival-predicting model was made specifically for patients with glioma to predict its survival and immune response to ICIs. C-MET was screened and validated for its tumor driver gene and immune regulation function (inducing t-cell exhaustion) in glioma.

## 1 Background

Regulated cell death (RCD) refers to a controlled and orderly type of cellular death (1, 2). The subtypes of these death modalities have been enriched with more and more RCDs uncovered, for instance, apoptosis (3–5), autophagy (6–8), necroptosis (9), ferroptosis (10), pyroptosis (11), cuproptosis (12), disulfidptosis (13), etc. Disulfidptosis is the latest type of RCD proposed in 2023 by Gan et al. (13). What distinguishes it from other forms of cell death is the feature that the aberrant accumulation of disulfides without enough nicotinamide adenine dinucleotide phosphate (NADPH) supply from glucose can induce this specific cell death (13–17). Disulfidptosis holds potential as an alternative therapeutic tactic for patients resistant to existing therapies.

Cancer is a notoriously formidable disease that is characterized by abnormal growth and division. Many types of cancer can metastasize to surrounding tissues or even distant organs. Until now, 14 hallmarks of cancer have been discovered, which have been summarized well by Douglas Hanahan (18). Resisting cell death, as one of the classical hallmarks, is always the fundamental and final objective for all other hallmarks. With each discovery of an innovative modality of cell death from apoptosis to cuproptosis, our understanding of cancer will be expanded further in that perspective. Numerous RCD-related prognostic signatures have been made and validated by different researchers. In the recent decade, ferroptosis (19, 20), pyroptosis (21–23), cuproptosis (24–27) have been well-explored in many types of cancer based on the cancer genome atlas (TCGA), gene expression omnibus (GEO), international cancer genome consortium (ICGC), etc. These studies give us a deeper understanding of RCD in the context of cancer.

Machine learning (ML), a subdomain of artificial intelligence (AI), can be divided into supervised, unsupervised, and reinforcement learning. In the era of big data, it can be applied everywhere (28, 29). And in oncology, ML techniques have also been employed to gain insights into the complex interactions between tumors and the immune system. For instance, in lymphoma, artificial neural networks were taken advantage of to construct an immune-oncology panel to differentiate molecular subtypes and predict prognosis (30). In solid tumors, ML-assisted analysis based on genomics or radiomics also gives us better models to identify treatment success rates (31–34).

However, to our knowledge, there are only limited studies on disulfidptosis. Given the lack of research on this phenomenon, our study aimed to delve into the role of disulfidptosis in pan-cancer relying on well-recognized databases by constructing a prognostic signature related to disulfidptosis. We mainly focused on investigating the crosstalk between disulfidptosis and tumor immune responses.

## 2 Methods

### 2.1 Data collection

Clinical features and gene expression of TCGA, ICGC, and PCAWG patients were obtained in UCSC Xena (http://xena.ucsc.edu). The validated transcriptomic data and clinical characteristics from glioma were fetched from CGGA (http://www.cgga.org.cn). The external gene expression and prognosis datasets of LUAD, UVM, and HNSC (GSE30219, GSE31210, GSE37745, GSE50081, GSE22138, GSE41613) were downloaded from GEO (https://www.ncbi.nlm.nih.gov/geo/). DRGs (ACTB, TLN1, CAPZB, STN, FLNB, IQGAP1, ACTN4, MYL6, FLNA, MYH9, MYH10, PDLIM1, CD2AP, and INF2) were extracted from Gan et al.’ disulfidptosis paper (13). Different immune cell infiltration markers were obtained from the cancer immunome atlas (TCIA) (35), Genecard (https://www.genecards.org/), GEPIA (http://gepia2.cancer-pku.cn/#index), Cibersot (https://cibersortx.stanford.edu/). The prognosis of different c-MET level glioblastoma patients treated with anti-PD1 therapy was obtained from Kaplan Meier-plotteR (http://kmplot.com/analysis/index.php?p=background).

### 2.2 Bioinformatic analysis

#### 2.2.1 Pathway score calculation and immune cell infiltration

ssGSEA was used to assess immune activity, function, and programmed cell death pathways in each sample. Immune cell marker genes were used for analysis. ESTIMATE calculated immune, stromal, estimate scores, and tumor purity based on immune and stromal cell proportions. TIMER and CIBERSORT predicted infiltrating immune cell composition. Immune checkpoint inhibitors were compared across clusters and risk groups. By analyzing ssGSEA, ESTIMATE, immune cell infiltration, and immune checkpoints, we gained a comprehensive understanding of the tumor immune landscape. Infiltration immune cell fractions were calculated in CIBERSORT in R4.2.0, and the estimate package in R4.2.0 predicted the immune score.

#### 2.2.2 Prognosis model construction

Univariate Cox regression, LASSO regression, and multivariate Cox regression were used to construct the gene signature. The previous survival and ROC analyses were made using survival and survivalROC packages in R4.2.0.

#### 2.2.3 DRGs-based subgroups identification

ConsensusClusterPlus package in R4.2.0 was used to perform consensus clustering analysis based on the DRGs (parameter: maxK=10, reps=50). AI modeling for DRGs-based prognosis model was developed by six AI functions, including extreme gradient boosting (XGboost, xgboost package in R4.2.0), support vector machine (SVM, e1071 packages in R4.2.0), multi-logistic (nnet packages in R4.2.0), random forest (RF, randomForest package in R4.2.0), deep learning (DL, h2o package in R4.2.0) and K-Nearest Neighbor (KNN, kknn package in R4.2.0). During the model construction, randomly select 75% as the training cohort and randomly select 25% as the testing cohort. Gene expression value was standardized to range “0~1” with preProcess function (caret and tidyverse packages).

#### 2.2.4 Tumor mutation analysis

We analyzed somatic mutations in TCGA data using “maftools” and calculated TMB for each group. Furthermore, we visualized somatic mutations of selected genes in the signature using cBioPortal. This analysis helped understand mutations and their potential role in disulfidptosis.

#### 2.2.5 Drug sensitivity prediction

Drug sensitivity prediction was performed by the oncoPredict package in R4.2.0. This package leverages machine learning algorithms trained on large datasets of cancer cell lines to estimate the response of individual patient tumors to a wide range of therapeutic agents. By analyzing the gene expression profiles of the tumor samples, oncoPredict can identify potential therapeutic targets and guide personalized treatment strategies.

### 2.3 Biological experiments

#### 2.3.1 Cell culture and reagents

Ln299 and Jurkat cell lines were purchased from the Chinese Academy of Science cell bank with STR matching analysis. Cells were cultured in recommended conditions. Co-culture was done by placing the transwell containing Jurkat cells (2.5 × 10^5^) or alive PBMC (2.5 × 10^5^) in the 6-well plate seeded with ln299 cells (20 x 10^4^). Cabozantinib (BMS-907351) was purchased from Selleck.

#### 2.3.2 SiRNA transfection

Ln299 cells were transfected with c-MET small interfering RNA (siRNA) (5′-AAG GAC CGG UUC AUC AAC UUC-3′) or non-targeting negative control siRNA (RiboBio, China) using LipofectamineTM 3000 (Invitrogen, USA) according to the manufacturer’s protocol.

#### 2.3.3 5-ethynyl-2′-deoxyuridine and live/dead staining

The live/dead staining kit was purchased from YEASEN Biotech, the Edu staining kit was purchased from APExBIO (K1077), and OPTI-MEM was purchased from (ThermoFisher, Gibco). 1×10^5^ ln299 cells were seeded into 24-well plates. The treated cells were stained according to the kits’ instructions and then observed under an inverted microscope.

#### 2.3.4 Western blotting

Total cellular proteins were extracted using lysis buffer (5 mM EDTA, 300 mM NaCl, 0.1% NP-40, 0.5 mM NaF, 0.5 mM Na3VO4, 0.5 mM PMSF, and 10 μg/mL each of aprotinin, pepstatin, and leupeptin; Sigma-Aldrich). 30–50 μg protein was separated using 10% SDS-PAGE and transferred to polyvinylidene difluoride membranes (Millipore, Bedford, MA, USA). Then immunoblotting was performed using antibodies against c-MET (25869-1-AP, Proteintech), PD-L1 (28076-1-AP, Proteintech), p-JAK3 (29101-1-AP, Proteintech), JAK3 (80331-1-RR, Proteintech), p-STAT3 (#9145, Cell Signaling Technology), STAT3 (#9139, Cell Signaling Technology), GAPDH (AF7021, Affinity Biosciences), IL-2 (16806-1-AP, Proteintech), INF-γ (15365-1-AP, Proteintech), PD1 (18106-1-AP, Proteintech), beta-tubulin (10068-1-AP, Proteintech). The immunoblots were visualized using an enhanced chemiluminescence detection system (Amersham Pharmacia Biotech, Uppsala, Sweden).

#### 2.3.5 PBMCs extraction

Simply, PBMCs were isolated via Ficoll-Paque density gradient centrifugation: 5 mL of peripheral blood was collected from healthy female volunteers, diluted with PBS at a 1:1 ratio, followed by gentle mixing. Add 10 mL of the diluted blood to 2 mL of Ficoll liquid (density 1.077). The clear stratification of blood and Ficoll liquid confirmed success. Carefully transferred the sample to the centrifuge and spin at 500 g for 15 minutes. Removed the centrifuge tube with care, aspirate the white thin film layer in the middle, representing individual nucleated cells. Wash the isolated nucleated cells with 10 mL of PBS, centrifuge at 250 g for 10 minutes, and discarded the supernatant. Repeat the washing step once and the suspended cells were frozen in vials at 100 million cells/mL in HI FBS with 5% DMSO after washing. Stored in liquid nitrogen, they were revived gradually and washed in pre-warmed RPMI with FBS and pen/strep. Following a 4-5 hour incubation at 37°C, viability was assessed using Trypan blue (0.1%).

#### 2.3.6 Flow cytometry

The co-cultured PBMC were stained with Fixable Viability Stain (Thermo, L34965) and Fc receptor blocking reagent [Ultra-LEAF™ Purified anti-mouse CD16/32 (101320, BioLegend)]. Next, they were stained with CD-3 (BD 557943), PD-1 (BD 561273), and CD8 antibody (thermo, A15448). The prepared single-cell suspensions were filtered through 40-μm nylon meshes (352340, Corning). Results were then acquired using BD Calibur, BD Fortessa, or Miltenyi MACSQuant systems. Data were analyzed with FlowJo_V10 software (TreeStar).

#### 2.3.7 ELISA

Supernatants from PBMC co-cultured with glioma cell line were collected and analyzed using ELISA kits for IL2(Proteintech, KE00017), IFN-γ (Proteintech, KE00146), CXCR9 (Proteintech, KE00165). The levels of each cytokine were compared between the c-MET knockdown group and control groups.

### 2.4 Statistical analysis

Statistical analyses were performed with R (4.2.0) and GraphPad Prism (version 8.0.1). Discontinuous data were expressed as numbers/percentages, and continuous data were expressed as mean ± standard deviation (SD). P < 0.05 was considered a statistically significant difference.

## 3 Results

### 3.1 The expression landscape and prognosis significance of DRGs in pan-cancer

**Figure 1:**
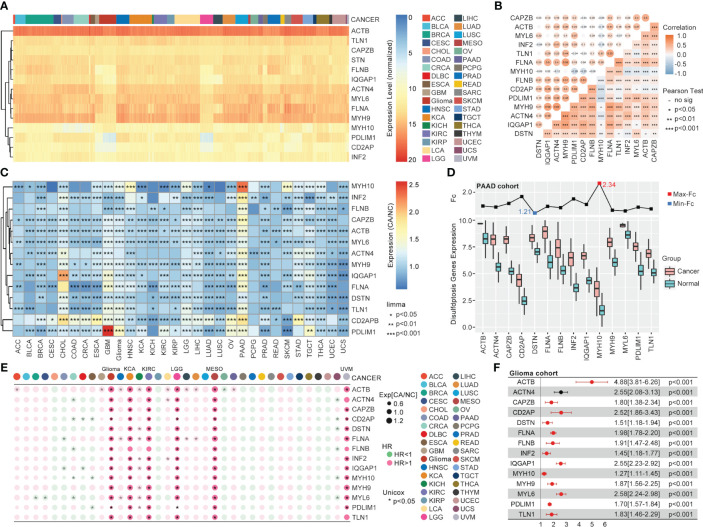
The pan-cancer landscape of DRGs. **(A)** The expression of 14 validated DRGs in all types (36) of cancer from TCGA. **(B)** The expression correlation analysis of DRGs, in which no significance of correlation was observed between MYH9 and MYH10, DSTN and TLN1, CD2AP and MYL6, IQGAP1 and MYL6, DSTN and ACTB. **(C)** The expression difference of DRGs between tumor samples (TCGA) and non-tumor samples (para tumor from TCGA + normal tissues from GTEx) in each type of cancer, expression difference existed in all DRGs in GBM, PAAD, PRAD, and TGCT. **(D)** The expression comparison between glioma tissues from TCGA and normal brain tissues from GTEx. **(E)** Univariate Cox regression analysis of DRGs in each type of cancer. **(F)** Univariate Cox regression analysis of DRGs in glioma, in which all DRGs were risk factors in glioma (HR>1, P<0.001).

In TCGA, the 14 validated disulfidptosis-related genes (DRGs) - ACTB, TLN1, CAPZB, STN, FLNB, IQGAP1, ACTN4, MYL6, FLNA, MYH9, MYH10, PDLIM1, CD2AP, and INF2 - were generally expressed in all 33 types of cancer (**Figure 1A**). The correlation analysis between the DRGs indicated that MYH9 and ACTN4 were the most positively related gene pair, while MYH10 and PDL1M1 were the most negatively related (**Figure 1B**). And the DRGs’ expression comparison between tumor and nontumor tissues implied a significant difference in most types of them (**Figure 1C**). MYH10 showed the highest 2.34-fold change between glioma and normal brain tissues among all the DRGs (**Figure 1D**). Moreover, the univariate Cox regression of the DRGs showed that almost all 14 DRGs could predict prognosis well in patients with glioma, kidney carcinoma (KCA), kidney renal clear cell carcinoma (KIRC), etc. (**Figure 1E**). Interestingly, DRGs were the completely hazardous factors in glioma (**Figure 1F**).

### 3.2 The correlation between immunity and disulfidptosis in pan-cancer

**Figure 2:**
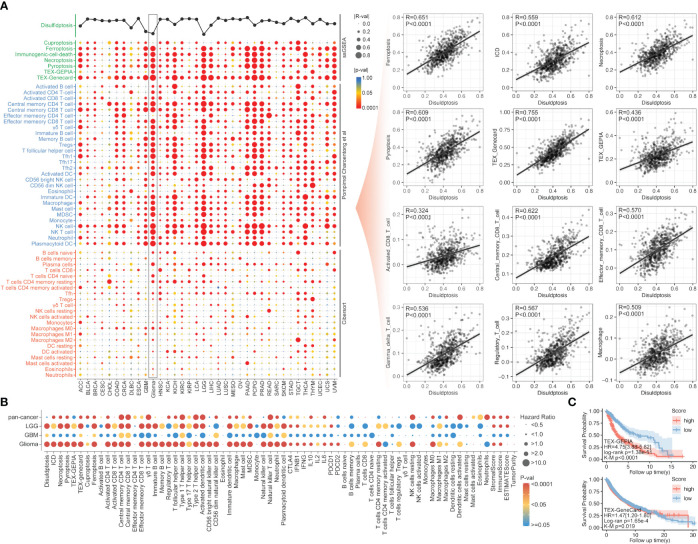
The correlation of immunity and other PCDs with disulfidptosis. **(A)** The correlation analysis between disulfidptosis and immune cell infiltration/other PCDs, in which disulfidptosis score was positively correlated with PCDs, including ferroptosis, ICD, necroptosis, and pyroptosis, and disulfidptosis was positive correlated with TEX, CD8+ T cells. **(B)** The univariate Cox regression of disulfidptosis, immune cell infiltration, and other PCDs in glioma, LGG, GBM, and pan-cancer. **(C)** The Kaplan–Meier survival analysis of Tex_GEPIA and Tex_GeneCard in pan-cancer, a higher score of both parameters was accompanied by worse prognosis in glioma (p<0.0001) evaluated by K-M analysis or unicox regression analysis.

Following the ssGSEA analysis of different immune cell infiltration and programmed cell death, the correlation analysis indicated a strong association between disulfidptosis and most immune cells. For the most significant glioma, the R-value between disulfidptosis and exhausted T cells (TEX_Genecard), central memory CD8 T cell, effector memory CD8 T cell, gamma delta T cell, regulatory T cell, macrophage was over 0.5 (**Figure 2A**). Interestingly, the correlation between disulfidptosis and other modalities of cell death like ferroptosis (R-value = 0.651), necroptosis (R-value = 0.612), pyroptosis (R-value = 0.609), immunogenic cell death (ICD) (R-value = 0.559) are also very high in glioma compared with other types of cancer (**Figure 2A**). The univariate Cox regression indicated that T cell exhaustion (Tex), immature B cell infiltration, etc., were the dangerous factors in glioma patients. In contrast, the activated NK cells’ infiltration was a beneficial factor for survival (**Figure 2B**). More importantly, a higher T cell exhaustion (TEX_GEPIA or TEX_Genecard) could predict a lousy prognosis in the glioma cohort from TCGA (**Figure 2C**).

### 3.3 Gene signature construction based on disulfidptosis for prognosis of patients with cancer

**Figure 3:**
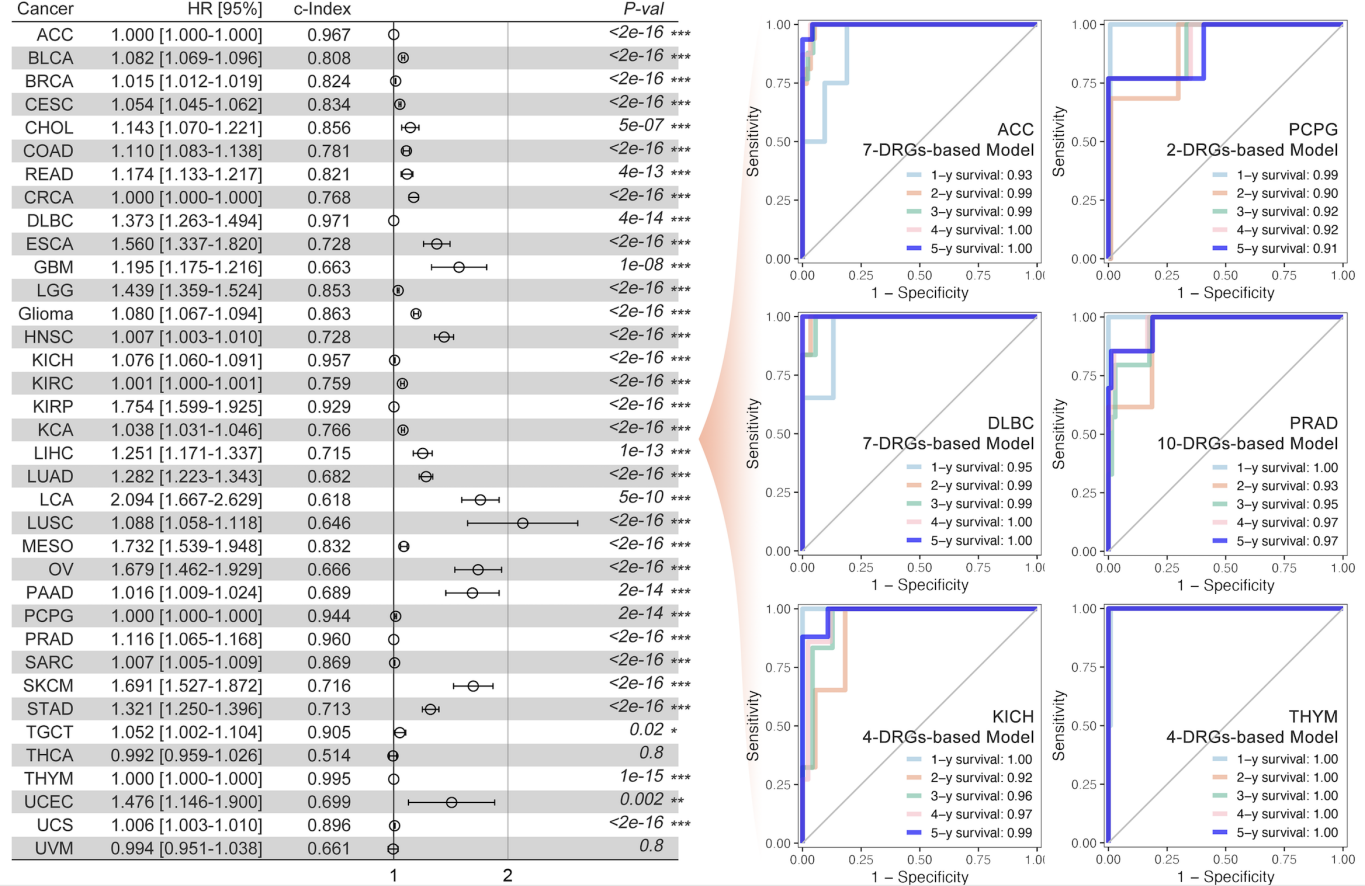
DRGs-based prognosis model and ROC curve. The DRGs-based gene signature for prognosis was constructed for each type of cancer (the left part), and the multi-gene-based model index was greater than 0.9 in ACC, DLBC, KICH, KIRP, PCPG, THYM, and TGCT. Multi-gene-based models for all cancer types were significantly constructed. The 1-year, 2-year, 3-year, 4-year, and 5-year ROC curve of the abovementioned gene signature was made for patients with ACC, PCPG, DLBC, PRAD, KICH, and THYM, respectively (the right part). * p<0.05, **p<0.01, ***p<0.001.

**Figure 4:**
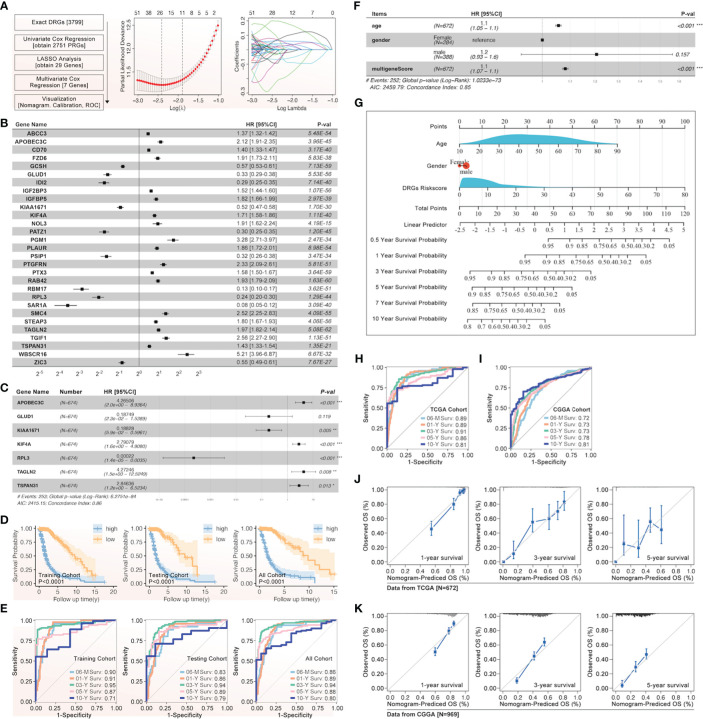
The gene signature of prognosis based on DRGs in glioma. **(A)** The flow chart and the LASSO regression results were listed, after which 29 genes were screened out, and **(B)** their effect on the prognosis of glioma was evaluated by univariate Cox, attached with HR and p-value. **(C)** The gene signature of glioma prognosis was made by multivariate Cox regression, in which APOBEC3C, GLUD1, KIAA1671, KIF4A, RPL3, TAGLN2, and TSPAN31 were input into the model. **(D)** The Kaplan–Meier curves were made in the training, testing, and all glioma cohorts from TCGA, and all displayed a similar result that a higher risk score was accompanied by a worse prognosis in glioma. **(E)** The ROC curves of 0.5-year, 1-year, 3-year, 5-year, and 10-year were presented in the training, testing, and all glioma cohorts from TCGA. **(F)** The gene signature based on DRGs and clinical characteristics for glioma were shown with HR value, in which age, gender, and multi-gene-based risk score were input into the model. **(G)** The glioma nomogram of gene signature based on DRGs and clinical characteristics. The glioma ROC curve of gene signature based on DRGs and clinical characteristics in TCGA **(H)** and CGGA **(I)**. The glioma nomogram prediction of gene signature based on DRGs and clinical characteristics in TCGA **(J)** and CGGA **(K)**. *p<0.05, **p<0.01, ***p<0.001.

The univariate Cox regression, least absolute shrinkage and selection operator (LASSO) regression, and multivariate Cox regression were used to construct a gene signature for each type of cancer. Except for thyroid cancer (THCA) and uveal melanoma (UVM), the gene signatures that could predict the prognosis for patients with all other types of cancer, respectively, were successfully made (**Figure 3**). For the top 6 gene signatures ranked by c-index, i.e., the gene signature in adrenocortical carcinoma (ACC), pheochromocytoma and paraganglioma (PCPG), lymphoid neoplasm diffuse large B-cell lymphoma (DLBC), prostate adenocarcinoma (PRAD), kidney chromophobe (KICH), and thymoma (THYM), the receiver operating characteristic (ROC) curves showed a very high area under the curve (AUC) for 1-year, 2-year, 3-year, 4-year, and 5-year survival (**Figure 3**). And in glioma that showed the most outstanding relation between disulfidptosis and immune cell infiltration (**Figures 2A, B**), its 7-gene signature (risk score = 1.56709174 * APOBEC3C + (-3.2556028) * GLUD1 + (-2.0800874) * KIAA1671 + 1.08729963 * KIF4A + (-7.9141641) * RPL3 + 1.83720741 * TAGLN2 + 1.89252831 * TSPAN31) (**Figures 4A–C**) was further validated by dividing the TCGA cohort into a training group and a testing group. And both the Kaplan–Meier (KM) analysis and ROC curve (0.5-year, 1-year, 3-year, 5-year, and 10-year) indicated significant results in the training group, testing group, and the whole group (**Figures 4D, E**). Then, the multivariant Cox analysis of the gene signature and the clinical characteristics implied that the gene signature was an independent hazard factor for the prognosis of patients with glioma (**Figure 4F**). The nomogram indicated the relation of age, gender, DRGs gene signature, and the survival probability (0.5-year, 1-year, 3-year, 5-year, 7-year, and 10-year) for glioma patients (**Figure 4G**). Furthermore, the model based on age, gender, and DRGs gene signature was validated in the Chinese Glioma Genome Atlas (CGGA) with AUC over 0.72 (**Figure 4I**). In both glioma patients from TCGA and CGGA, there was a consistency between the predictive model and survival rate in the real world (**Figures 4J, K**).

### 3.4 Unsupervised pan-cancer clustering analysis based on DRGs and tumor mutation burden comparison

**Figure 5:**
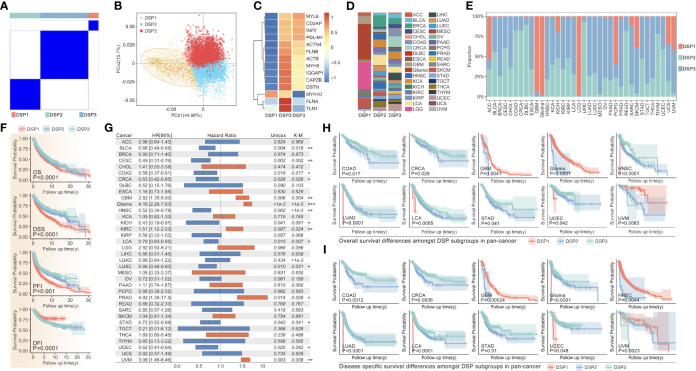
DRGs-based clustering and prognosis analysis in pan-cancer. **(A)** The unsupervised clustering of DRGs in pan-cancer based on the 14 DRGs (MYL6, CD2AP, INF2, PDLIM1, ACTN4, FLNB, ACTB, MYH9, IQGAP1, CAPZB, DSTN, MYH10, FLNA, TLN1). **(B)** PCA analysis shows the sample distribution amongst subgroups (DSP1, DSP2, DSP3). **(C)** DRGs expression profile feature in subgroups. **(D)** Tumor sample distribution amongst subgroups. **(E)** Subgroup distribution proportion in 36 kinds of cancer. **(F)** OS, DSS, PFI, and DFI analysis among different DSP groups in pan-cancer were all significant (p<0.001). **(G)** The univariate Cox regression (OS) of DSP clusters in every type of cancer from TCGA, in which significance was observed in BLCA, CESC, COAD, CRCA, Glioma, HNSC, KICH, KIRC, LCA, LUAD, LUSC, PRAD, STAD, UCEC and UVM. OS analysis **(H)** and DSS analysis **(I)** in COAD, CRCA, GBM, glioma, HNSC, LUAD, LCA, STAD, and UCEC. * p<0.05, **p<0.01, ***p<0.001.

**Figure 6:**
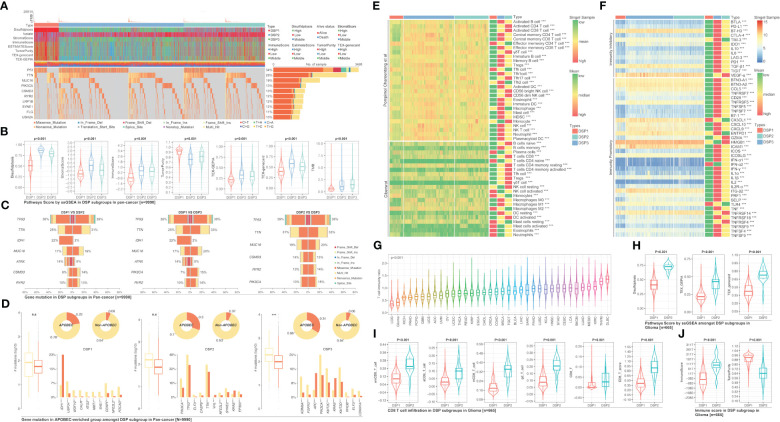
Gene mutation comparison among DSP groups in pan-cancer. **(A)** Gene mutation landscape among DSP groups in pan-cancer. **(B)** Pathways score in DSP groups in pan-cancer. **(C)** Mutation comparison between every two DSP groups. **(D)** Mutation comparison between APOBEC-enriched and non-APOBEC-enriched patients in each DSP group. Immune cell infiltration **(E)** Immune cell infiltration in DSP group, **(F)** Immunocheck points expression in DSP groups. **(G)** Immune score status in 36 types of cancer. **(H)** Disulfidptosis score, TEX_GEPIA, and TEX_gencard were higher in DSP2 in glioma (p<0.001). **(I)** Various types of CD8+ T cells infiltration differences in DSP groups in glioma (p<0.001). **(J)** Immune score and tumor purity differences in DSP groups in glioma (p<0.001). **p<0.01, ***p<0.001; ns, significant.

The unsupervised clustering analysis based on the 14 DRGs’ expression was used to categorize the TCGA cohort into disulfidptosis (DSP)1, DSP2, and DSP3 groups (**Figures 5A–E**). The KM analysis suggested the DSP groups had significantly different survival in the disease‐free interval (DFI), disease‐specific survival (DSS), overall survival (OS), and progression‐free interval (PFI) (**Figure 5F**). In line with the KM analysis of pan-cancer, the KM analysis or univariate Cox regression in individual cancer type indicated that the 3 DSP clusters could serve as a significant survival-related factor in colon adenocarcinoma (COAD), CRCA [COAD + rectum adenocarcinoma (READ)], glioblastoma multiforme (GBM), glioma, head and neck squamous cell carcinoma (HNSC), kidney chromophobe (KICH), kidney renal clear cell carcinoma (KIRC), lung adenocarcinoma (LUAD), lung carcinoma (LCA), stomach adenocarcinoma (STAD), uterine corpus endometrial carcinoma (UCEC), and uveal melanoma (UVM) (**Figures 5G–I**). Next, the top 10 mutated genes (TP53, TTN, MUC16, etc.) were listed and compared among DSP1, DSP2, and DSP3 groups (**Figures 6A, C**). Besides, the disulfidptosis, stromal score, immune score, tumor purity, Tex, and tumor mutation burden (TMB) were significantly different among the 3 DSP groups (**Figure 6B**). Since the previous 7-gene model included APOEBC3C, the TMB between APOBEC-enriched and APOBEC-unenriched groups was also compared in each DSP group (**Figure 6D**). Immune cell infiltration and immune molecules differed greatly among the 3 DSP groups (**Figures 6E, F**). Each cancer type’s total T-cell infiltration ratio was also listed to give a whole landscape (**Figure 6G**). In particular, the glioma, in which DRGs models showed the most significant relationship with survival and immunity, implicated a significant difference in disulfidptosis, Tex_GEPIA, Tex_genecard, CD8 (+) T cell subtypes, immune score, and tumor purity between the two DSP subgroups (**Figures 6H–J**).

### 3.5 Refined DSP models construction and validation by WGCNA and machine learning in pan-cancer

**Figure 7:**
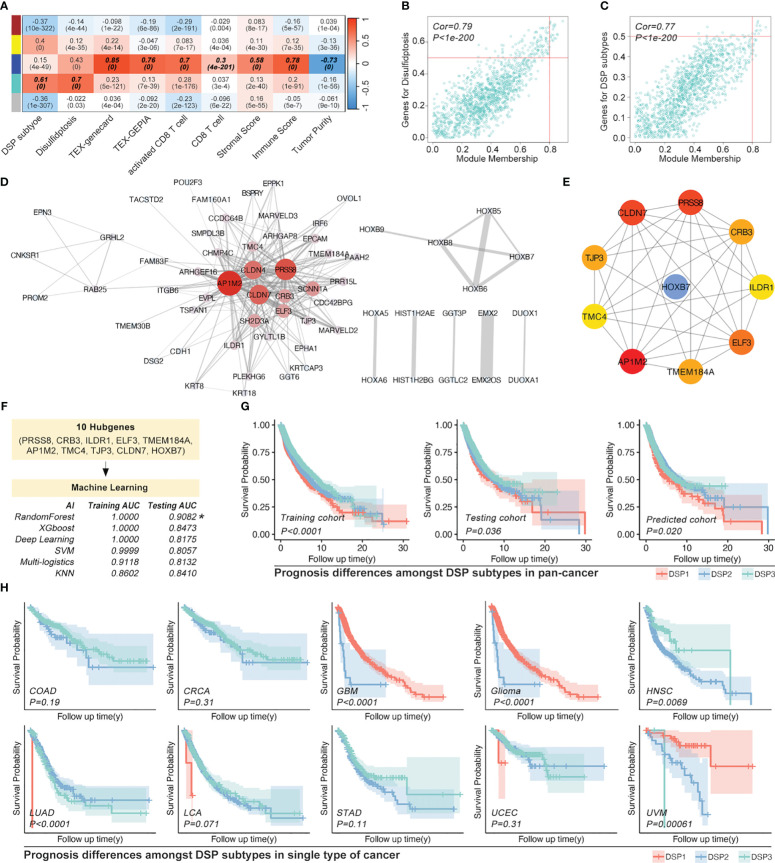
Refined prognostic model construction in pan-cancer by WGCNA and Machine learning. **(A)** Gene modules correlated with DSP pathways and immune cell infiltration by WGCNA, in which **(B, C)** module gene cohorts were most linked with DSP grouping and disulfidptosis (Cor=0.79, p<1e-200), while deep blue module gene cohorts were most correlated with immune cell infiltration (Cor=0.77, p<1e-200). **(D)** Gene interaction network about top 50 DSP grouping related genes in cyan module gene cohorts **(E)** Hub genes of the cyan gene module. **(F)** Refined prognostic model construction based on pan-cancer by supervised machine learning, in which random forest algorithm displayed as the most efficient (Training AUC=0.9082). **(G)** K-M analysis indicated the prognosis differences amongst DSP groups in the training cohort, testing cohort (original groups), and predicted group (AI-identified group using test cohort data). **(H)** Refined prognostic model performance in the OS analysis of COAD, CRCA, GBM, glioma, HNSC, LUAD, LCA, STAD, and UCEC.

**Figure 8:**
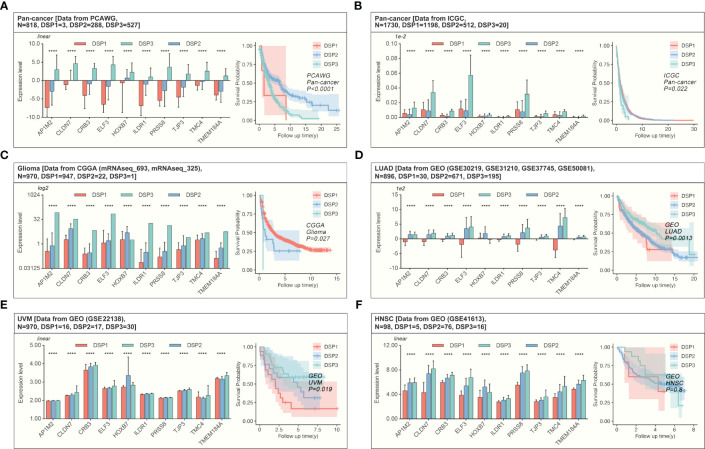
Validation of the refined prognostic model in external datasets. Expression of DRGs and validation of the refined prognostic model in pan-cancer from **(A, B)** PCAWG (p<0.0001) or ICGC (p=0.022), both of them showed significant prognosis differences in AI-identified DSP subgroups. **(C)** The Glioma cohort from CGGA manifested significant prognosis differences amongst AI-identified DSP groups (p=0.027). **(D)**, LUAD from GEO datasets (GSE30219, GSE31210, GSE37745, GSE50081) presented significant prognosis differences amongst AI-identified DSP groups (p=0.0013), **(E)** UVM from GSE22138 showed significant prognosis difference amongst AI-identified DSP groups (p=0.019) **(F)** HNSC from GSE41613 (exhibited insignificant prognosis difference amongst AI-identified DSP groups (p=0.8). ****p<0.0001.

The weighted correlation network analysis (WGCNA) was used to extract the gene module most associated with disulfidptosis, immune cell infiltration, etc. (**Figures 7A–C**). Next, the ten hub genes (PRSS8, CRB3, ILDR1, ELF3, TMEM184A, AP1M2, TMC4, TJP3, CLDN7, HOXB7) within this cyan module were further abstracted by the STRING database and cytoHubba (**Figures 7D, E**). The refined DSP models based on the ten hub genes were then constructed by employing the best method of machine learning-randomForest, in which the training and testing cohorts have the highest AUC (**Figure 7F**). Moreover, compared with the original DSP groups, it could better predict the prognosis in pan-cancer patients (**Figure 7G**). The refined DSP models could differentiate the prognosis more evidently in patients with glioma (**Figure 7H**). After that, the new DSP model was also validated in pan-cancer cohorts from PCAWG and ICGC, glioma from CGGA, LUAD from GEO (GSE30219, GSE31210, GSE37745, GSE50081), and UVM from GEO (GSE22138) with significant p-value (**Figures 8A–F**).

### 3.6 Enhanced refined DSP models construction in glioma

**Figure 9:**
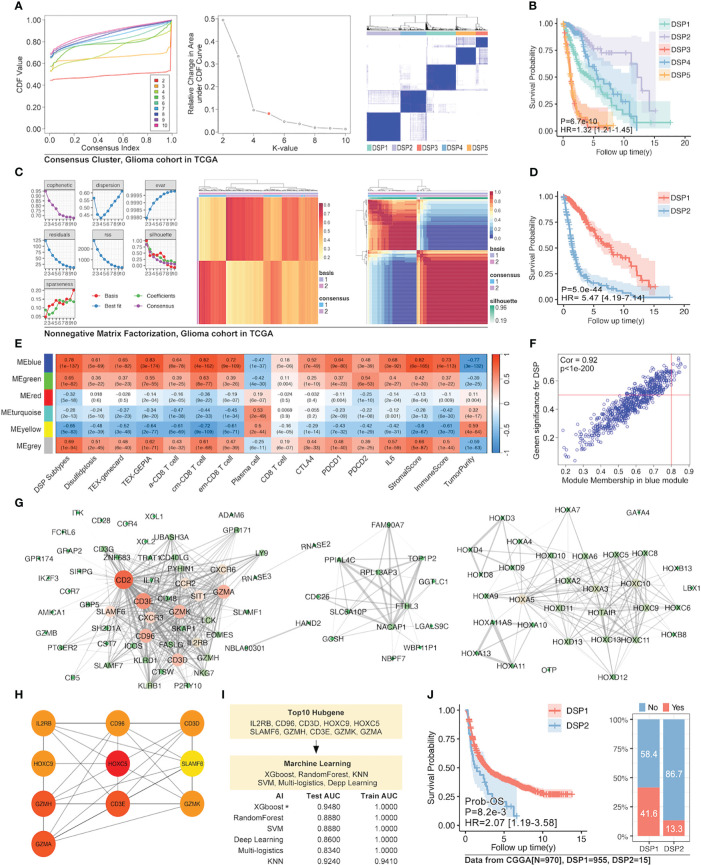
Enhanced prognostic model in glioma by WGCNA and machine learning. **(A)** Unsupervised consensus clustering of 14 validated DRGs **(B)** and its survival analysis in the glioma cohort, which displayed a significant difference in prognosis (p=6.7e-10). **(C)** The clustering of 14 validated DRGs by Non-negative Matrix Factorization (NMF) divided the glioma cohort into two groups with **(D)** significantly different prognoses (p=5e-44). **(E)** WGCNA for NMF clustering DSP groups, in which blue module gene cohort was the most correlated to DSP grouping, immune cell infiltration, and immunecheckpoint expression (p<0.0001). **(F)** The correlation analysis of the blue gene module from WGCNA and DSP subtypes. The blue gene module **(G)** and its hub genes **(H)** network. **(I)** Enhanced prognostic model based on hub genes for patients with glioma by machine learning, among which the xgboost algorithm showed the best accuracy (testing AUC=0.9480). **(J)** The validation of the enhanced prognostic model in glioma patients from CGGA by KM analysis and immune checkpoint inhibitors response prediction (p<0.001).

Since the refined DSP model performed exceptionally well in glioma among all the types of cancer, the unsupervised consensus clustering and non-negative matrix factorization (NMF) clustering were further utilized to categorize the DRGs into different groups (**Figures 9A, C**). Finally, the more practical and evident two-DSP-group classification by the NMF method was chosen for further construction of gene signature. Compared with a lack of significance between the survival of some subtypes by the consensus clustering (**Figure 9B**), the KM analysis indicated a significant difference (p < 0.0001) between DSP1 and DSP2 with Hazard Ratio (HR) equal to 5.47 (**Figure 9D**). Furthermore, the blue module, most correlated with DSP subtypes classification and immune cell infiltration, was extracted by WGCNA (**Figures 9E–G**). Ten hub genes (IL2RB, CD96, CD3D, HOXC9, HOXC5, SLAMF6, GZMH, CD3E, GZMK, and GZMA) from this module were screened by cytoHubba to construct an enhanced refined DSP clustering model by ML in glioma (**Figure 9H**). Surprisingly, the glioma-customized DSP model trained from TCGA could predict survival well in the glioma cohort from CGGA (**Figures 9I, J**). Moreover, The DSP1 has a 3-fold immune therapy response rate than the DSP2 group by oncoPredict package prediction (R.4.2.0).

### 3.7 The c-MET mechanism exploration by experiments

**Figure 10:**
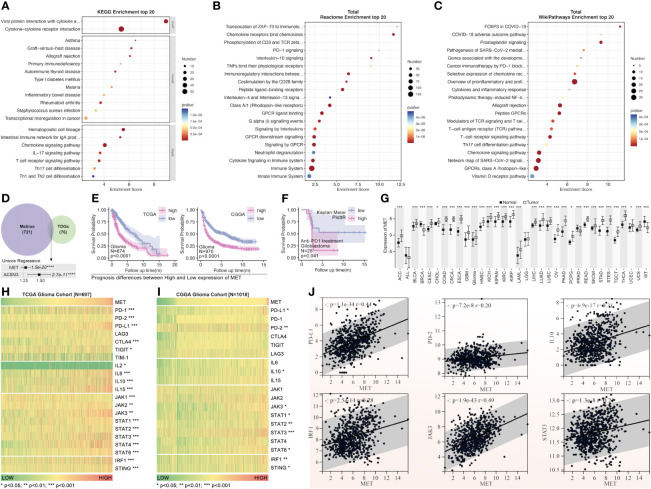
The pathway enrichment and tumor driver genes analysis from the blue gene module. Pathway enrichment of blue gene module by KEGG **(A)**, Reactome **(B)**, and WikiPathways **(C)**. **(D)** The tumor driver genes’ extraction from the blue module. **(E)** The c-MET survival analysis of patients with glioma from TCGA and CGGA (HR>1.25, p=1.5e-20). **(F)** The c-MET prognosis analysis was validated in the glioblastoma cohort receiving anti-PD1 treatment from “Kaplan-Meier Plotter” (http://kmplot.com/analysis/index). **(G)** The expression of c-MET in pan-cancer and non-tumor tissues(data from TCGA and GTEx). The immune markers expression was based on the c-MET expression in the glioma cohort from TCGA **(H)** and CGGA **(I)**. **(J)** The expression correlation analysis between different immune markers (PDL1, PD2, IL10, IRF1, JAK3, STAT3) and c-MET in the glioma cohort from TCGA. *p<0.05, **p<0.01, ***p<0.001, ****p<0.0001

**Figure 11:**
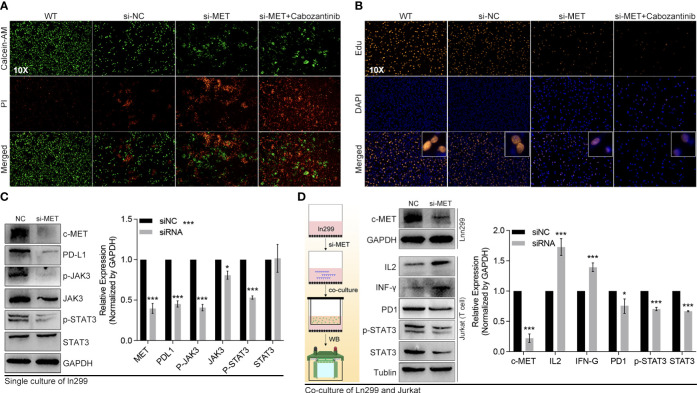
C-MET was a tumor driver gene and could inhibit the JAK3-STAT3 pathway. **(A)** The live and dead cell staining by Calcein and PI, in which siRNA-c-MET treatment increases the dead cell proportion induced by cabozantinib treatment. **(B)** The Edu and DAPI staining of the ln299 cell line. **(C)** The protein expression alteration after c-MET knockdown in the ln299 cell line, in which PDL1, p-JAK3, JAK3, and pSTAT3 were down-regulated, while **(D)** the expression of IL2 and IFN-γ were up-regulated in the Jurkat cell line in co-culture system. *p<0.05, ***p<0.001.

The pathway enrichment of the blue gene module implied that these genes might be involved in PD1 regulation (**Figures 10A–C**). The c-MET inspired us to explore its function further since it was one of the top 2 genes in both the blue module and tumor driver genes (TDG) (36) (**Figure 10D**). High expression of c-MET was associated with poor survival among glioma patients from TCGA and CGGA (**Figures 10D, E**). More importantly, the survival tendency in glioblastoma patients receiving anti-PD1 therapy agreed with the previous two cohorts (**Figure 10F**). Interestingly, its expression differed significantly between tumor and nontumor samples in over 90% of cancer types (**Figure 10G**). Interestingly, most immune markers in glioma had an expression difference between the high-c-MET and low-c-MET groups (**Figures 10H, I**). The expression of c-MET was positively linked with PD-L1, PD2, IL-10, IRF1, JAK3, and STAT3 (**Figure 10J**). Furthermore, the *in-vitro* experiment results indicated that the knockdown of c-MET could decrease the survival (**Figure 11A**) and proliferation (**Figure 11B**) of glioblastoma cell line ln299, which could be further enhanced by the combination treatment with cabozantinib (2μM, a c-MET inhibitor) (**Figures 11A, B**). In line with our previous data, the decrease of c-MET could down-regulated the p-JAK3, p-STAT3, and PD-L1 (**Figure 11C**). Furthermore, the Jurkat T cell co-cultured with the ln299 of c-MET knockdown obtained a higher level of IL-2, IFN-γ, and PD-1 (**Figure 11D**).

**Figure 12:**
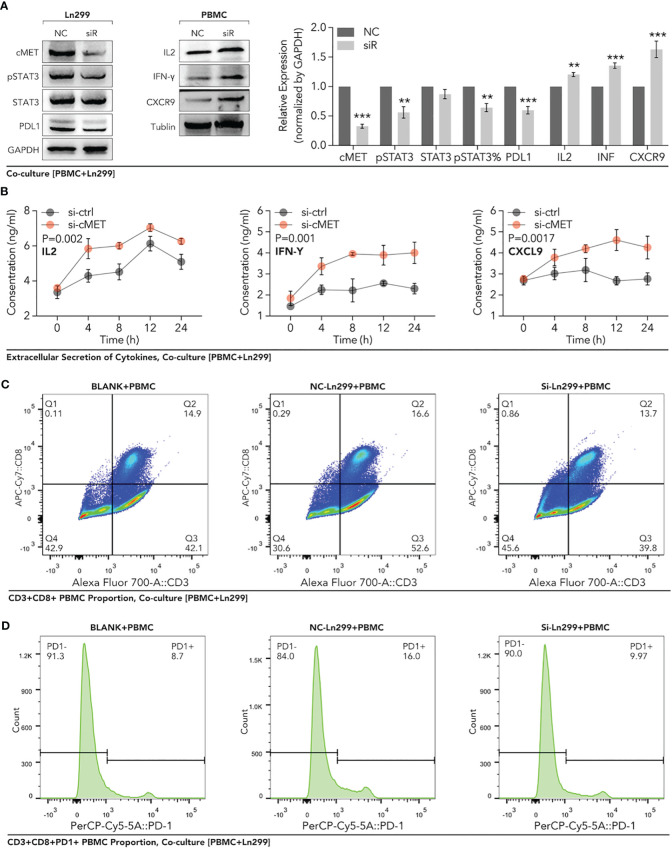
Down-regulation of c-MET within glioma enhanced the PBMC-derived CD8+ T cell function and proportion in the co-culture system. Glioma cell line Ln299 cells were treated with c-MET siRNA for 24h and co-cultured with PBMC for another 24h. **(A)** WB was used to detect the relevant protein expression in Ln299 and PBMC, in which PDL1, STAT3, pSTAT3, and pSTAT3 were down-regulated in Ln299. At the same time, IL2, IFN-γ, and CXCR9 were up-regulated in PBMC. **(B)** ELISA was applied to detect extracellular protein levels in the co-culture system, in which IL2, IFN-γ, and CXCL9 were higher in the si-c-MET group than those in the NC group. **(C)** The proportion of PD1+ PBMC was decreased by the down-regulation of c-MET in ln299 a little. **(D)** PD1+ CD3+CD8+ T cells were reduced evidently in the si-c-MET group than those in the NC group. **p<0.01, ***p<0.001.

To further verify the regulation of c-MET on PD1/PDL1, peripheral blood mononuclear cells (PBMC) were extracted from healthy females. Through the co-culture of PBMC and glioma cells, our data showed that down-regulation of c-MET in Ln299 significantly decreased the activation of STAT3 and the expression level of PDL1 in this cell (**Figure 12A**). In contrast, the expression level of IL2, IFN-γ, CD8 and CXCR9 were elevated in PBMC (**Figure 12A**). Furthermore, extracellular level of IL2, IFN-γ, and CXCL9 were also significantly increased in the culture media (**Figure 12B**). Next, FACS was applied to detect the c-MET-mediated CD8+ T cell immunity inhibition. In **Figure 12C**, we found that the proportion of CD8+ T cells was increased a little after co-culture with glioma cells while it could return to normal level (**Figure 12C**). However, this phenomenon was very marginal compared with the PD1 change in CD8+ T cells. The CD3+ CD8+ T cells with high PD-1 expression elevated from 8.8% to 16% after co-cultured with ln299 cells. In contrast, the knockdown of c-MET almost reversed the T-cell exhaustion completely (**Figure 12D**).

## Discussion

Disulfidptosis was a new modality of programmed cell death coined by Gan et al. in 2023 (13), with very little further research on cancer immunity. Our study explored the DRGs’ role in 33 types of cancer in detail. The limma package and univariate Cox regression indicated that the 14 validated DRGs did not only manifest significantly different expressions between tumors and normal + para tumor tissues, but they could also predict differential survival in glioma, KCA, KIRC, MESO, and UVM (**Figures 1C, E**). In particular, each gene of the 14 DRGs could play a significant role in the prognosis of patients with glioma (**Figure 1F**). Although some genes in the DRGs had been reported to be involved in glioma, our results implicated how the disulfidptosis pathway is regulated by these genes in glioma deserves more research (37–42).

Besides other types of PCDs, the correlation analysis showed that the disulfidptosis was also closely related to immune cell infiltration, including Tex_Genecard, Tex_GEPIA, CD8 (+) T cells, regulatory T cells, and macrophages (**Figure 2A**). Our data even suggested that disulfidptosis-postively-related Tex by both gene cards and GEPIA was a harmful factor in the prognosis of glioma (**Figure 2B**). PCD of different cells in the tumor microenvironment (TME) has been found to complicate cancer therapy. On the one hand, evidence suggested that cancer cells undergoing PCD in TME might render them more difficult to survive (43–46). On the other hand, other immune components undergoing RCD in the TME could alter immune attacks on tumor cells. For instance, the necroptosis induced in the TME was reported to enhance the immune surveillance from the BATF3 (+) conventional dendritic cells 1 (cDC1) and CD8 (+) T cells, leading to the release of many immunostimulatory cytokines (47–51). However, necroptosis induction in pancreatic cancer was found to protect the tumor cell from attacks by immune cells (52). While pyroptosis could induce antitumor effects by increasing the infiltration of dendritic cells (DC), CD4 (+) T cells, and CD8 (+) T cells (53, 54). For ferroptosis, it was reported to promote immunogenicity, induce DCs’ phenotypic development, and elicit a vaccination-like response (55). The expression of cuproptosis-related genes was positively correlated with PD-L1 expression and negatively associated with regulatory T-cell infiltration in melanoma (56). To our knowledge, our study was the first to explore disulfidptosis and tumor immune infiltration in pan-cancer patients and gave a complete picture of disulfidptosis’ role in immune regulation.

Our study even constructed a rough gene signature based on disulfidptosis genes to predict the survival of all patients of every cancer from TCGA (**Figure 3**). In ACC, PCPG, DLBC, PRAD, KICH, and THYM, the DRGs-based model could predict 1-year, 2-year, 3-year, 4-year, and 5-year survival with over 0.9 AUC (**Figure 3**). The gene signature based on PCD-related genes has always been a popular research direction. However, there is still a lack of the DRGs-related prognostic gene signature (57–62). Our research is the first to make a gene signature for each type of cancer patient from TCGA. Moreover, we further analyzed the DRGs-based model in glioma in which Tex and immune cell infiltration was strongly associated with disulfidptosis (**Figure 2B**). In both the TCGA and CGGA glioma cohorts, the gene signature’s predictive effect was significant and consistent (**Figures 4D, E, H–K**). To further dissect the role of disulfidptosis in pan-cancer, we clustered the 14 validated DRGs by their expression pattern in pan-cancer. The three DSP groups had significantly different OS, DSS, PFI, and DFI in pan-cancer (**Figure 5F**). More importantly, DSP groups also had disparate DFI and OS in COAD, CRCA, GBM, glioma, HNSC, LUAD, LCA, STAD, UCEC, and UVM (**Figures 5F–I**). The consistent survival significance of DSP clustering indicated that this new form of PCD was important in these types of cancer. Further tumor mutation burden (TMB) analysis suggested that the TP53, TTN, and IDH1 mutations may be involved in the disulfidptosis. Despite the regulation on nearly all previously reported PCD by TP53, no studies have explored its role in disulfidptosis until now (63). Our data provided many possible candidates to uncover more mechanisms of disulfidptosis. Consistent with the previous immune cell infiltration analysis, our result showed that there was a higher Tex within the DSP2 than DSP1 in glioma patients (**Figure 6G**), which gave more evidence that disulfidptosis was closely linked with Tex (**Figure 6I**).

To further obtain a refined DSP model, WGCNA, followed by machine learning, was employed to explore the most relevant gene modules with disulfidptosis. Ten hub genes, including PRSS8, CRB3, ILDR1, ELF3, TMEM184A, AP1M2, TMC4, TJP3, CLDN7, and HOXB7, were extracted from the most related gene module (**Figure 7E**). Next, randomForest machine learning, dependent on the ten hub genes, produced the best prognosis model by virtue of categorizing different DSP groups in pan-cancer, which was even validated in external databases (**Figures 7G**, **8A–F**). Our study proposed a generally effective prognosis model for pan-cancer. Interestingly, it worked exceptionally well in glioma, LUAD, and UVM. Combined with the abovementioned results, it inspired us to continue analyzing disulfidptosis in glioma. A specific prognosis model for patients with glioma was constructed based on ten hub genes (IL2RB, CD96, CD3D, HOXC9, HOXC5, SLAMF6, GZMH, CD3E, GZMK, and GZMA) (**Figures 9H, I**). Glioma was divided into DSP1 and DSP2 groups, where the DSP1 group was predicted to have a much higher response rate to immune checkpoint inhibitors (ICIs) than the DSP2 group (**Figure 9J**).

Finally, our further mechanism exploration revealed that c-MET might play a vital role in the interaction between disulfidptosis and glioma immunity. The high expression of c-MET could even predict a poor prognosis in glioblastoma patients receiving anti-PD1 treatment (**Figure 10F**). This tumor driver gene also manifested a positive relation with the JAK3-STAT3-PD-L1 pathway (**Figure 10J**). JAK/STAT signaling is reported to play pivotal roles in tumor immunity, including the maintenance of activated T cells (64–68). This phenomenon was further validated in *in-vitro* experiments where we co-cultured the c-MET-knockdown glioblastoma cell line with the Jurkat T cell line (**Figures 11A–D**, **12A–D**). The promotion of cell death and inhibition of cell proliferation by c-MET knockdown indicated that it could serve as a tumor driver gene. Its regulation on JAK3-STAT3-PD1/PD-L1 in T cells indicated the crosstalk between disulfidptosis and T-cell exhaustion. Targeting c-MET by siRNA or cabozantinib might be a promising way to enhance the T cell function implicated by the decreased high-PD1 T cells proportion and the increased CXCR9, CXCL9, IL2, and INF-γ (**Figures 11D**, **12A–D**). Although we uncovered many potential and exciting candidates for further research on disulfidptosis and cancer immunity, more efforts are needed to validate their functions.

## Conclusions

To summarize, we dissected the expression of DRGs between cancerous and noncancerous tissues, their roles in the prognosis, and their relationship with immunity in pan-cancer. A general prognosis model based on machine learning was constructed for pan-cancer and validated by external datasets with a consistent result. In particular, a DSP prognosis model was made specifically for patients with glioma to predict its survival and immune response to ICIs. Many potential candidates were screened, among which c-MET was validated for its TDG and immune regulation roles (inducing t-cell exhaustion) in glioma.

## Data availability statement

The original contributions presented in the study are included in the article/**Supplementary Material**. Further inquiries can be directed to the corresponding authors.

## Ethics statement

The studies involving humans were approved by Medical Ethics Committee of The First People’s Hospital of Xiaoshan District. The studies were conducted in accordance with the local legislation and institutional requirements. The participants provided their written informed consent to participate in this study.

## Author contributions

PL: Funding acquisition, Software, Validation, Writing – original draft. SW: Data curation, Investigation, Methodology, Software, Writing – original draft. HW: Formal analysis, Investigation, Supervision, Validation, Writing – original draft. YH: Data curation, Formal analysis, Writing – original draft. KY: Methodology, Software, Writing – original draft. KS: Formal analysis, Investigation, Supervision, Writing – original draft. ZW: Funding acquisition, Project administration, Writing – review & editing. HJ: Formal analysis, Writing – review & editing, Funding acquisition, Project administration.

## Supplementary material

Supplementary Figure 1 Gating strategy for PBMC co-cultured with Ln299 cells. **(A)** The gating detail for PBMC only. **(B)** The gating detail for PBMC co-cultured with ln299 cell for 48h. **(C)** The gating detail for PBMC co-cultured with c-MET-knockdown ln299 cell for 48h.

## Glossary

**Table:**

ACC	Adrenocortical carcinoma
AUC	Area under the curve
AI	Artificial intelligence
CGGA	Chinese Glioma Genome Atlas
COAD	Colon adenocarcinoma
cDC1	Conventional dendritic cells 1
CRCA	COAD + rectum adenocarcinoma
DC	Dendritic cells
DFI	Disease-free interval
DSS	Disease-specific survival
DSP	Disulfidptosis
DRGs	Disulfidptosis-related genes
Edu	5-ethynyl-2′-deoxyuridine
GEO	Gene expression omnibus
GBM	Glioblastoma multiforme
HR	Hazard Ratio
HNSC	Head and neck squamous cell carcinoma
ICIs	Immune checkpoint inhibitors
ICD	Immunogenic cell death
ICGC	International Cancer Genome Consortium
KCA	Kidney carcinoma
KICH	Kidney chromophobe
KIRC	Kidney renal clear cell carcinoma
LASSO	Least absolute shrinkage and selection operator
LUAD	Lung adenocarcinoma
LCA	Lung carcinoma
DLBC	Lymphoid neoplasm diffuse large B-cell lymphoma
ML	Machine learning
NADPH	Nicotinamide adenine dinucleotide phosphate
NMF	Non-negative matrix factorization
OS	Overall survival
PBMC	peripheral blood mononuclear cells
PCPG	Pheochromocytoma and paraganglioma
PFI	Progression-free interval
PRAD	Prostate adenocarcinoma
ROC	Receiver operating characteristic
RCD	Regulated cell death
ssGSEA	Single-sample Gene Set Enrichment Analysis
siRNA	Small interfering RNA
STAD	Stomach adenocarcinoma
Tex	T cell exhaustion
TCGA	The Cancer Genome Atlas
TCIA	The Cancer Immunome Atlas
THCA	Thyroid cancer
THYM	Thymoma
TDG	Tumor driver genes
TME	Tumor microenvironment
TMB	Tumor mutation burden
UCEC	Uterine corpus endometrial carcinoma
UVM	Uveal melanoma
WGCNA	Weighted correlation network analysis
