# Supplementary figures and images for: Construction of disulfidptosis-based immune response prediction model with artificial intelligence and validation of the pivotal grouping oncogene c-MET in regulating T cell exhaustion
Source: Front Immunol. 2024 Jan 26;15:1258475. doi: 10.3389/fimmu.2024.1258475 (PMC10862485; doi:10.3389/fimmu.2024.1258475)

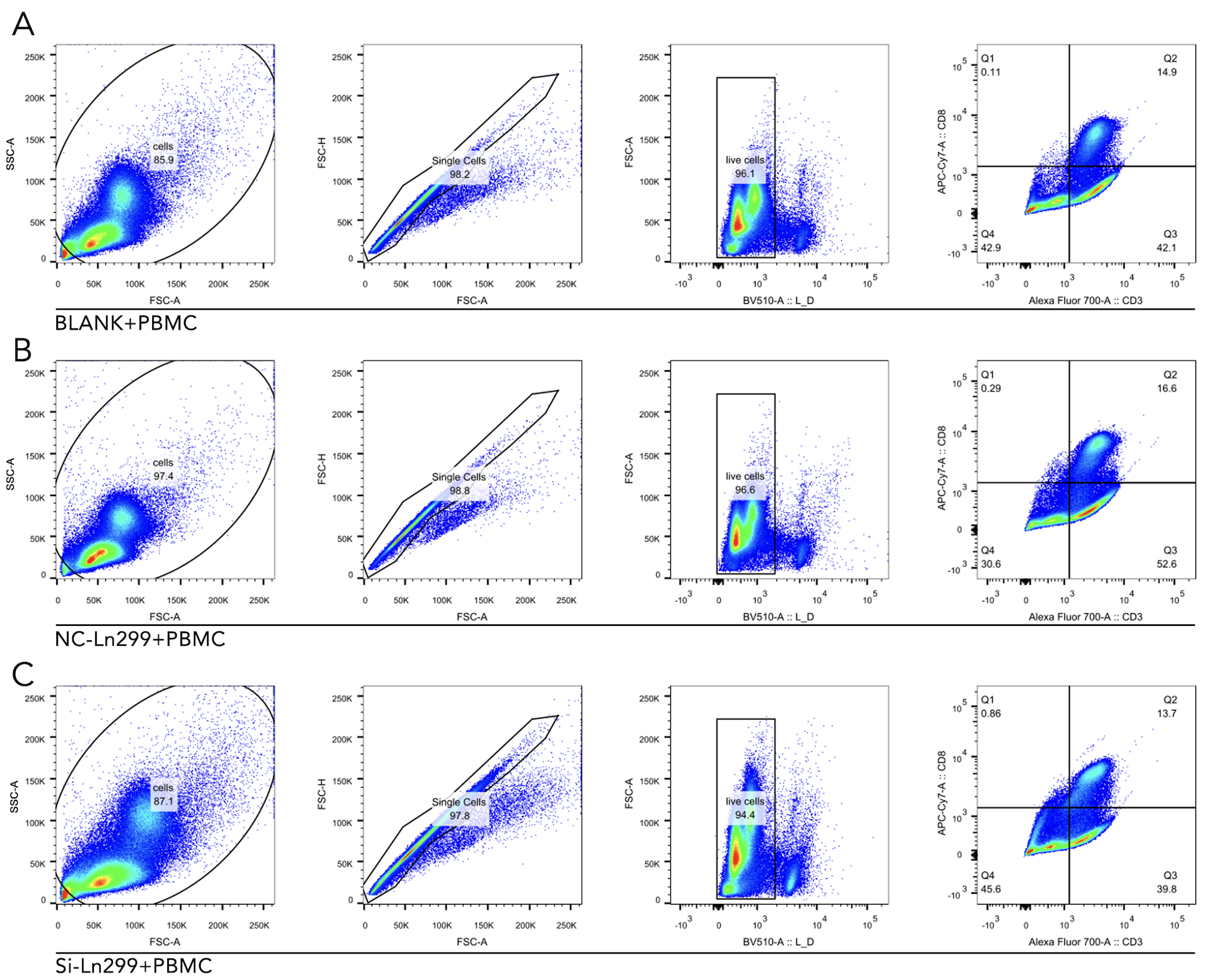

Supplement: Supplementary Figure 1 — Gating strategy for PBMC co-cultured with Ln299 cells. (A) The gating detail for PBMC only. (B) The gating detail for PBMC co-cultured with ln299 cell for 48h. (C) The gating detail for PBMC co-cultured with c-MET-knockdown ln299 cell for 48h. [file Image_1.png]
